# Need for ICU and outcome of critically ill patients with COVID-19 and haematological malignancies: results from the EPICOVIDEHA survey

**DOI:** 10.1007/s15010-023-02169-7

**Published:** 2024-02-22

**Authors:** Tobias Lahmer, Jon Salmanton-García, Francesco Marchesi, Shaimaa El-Ashwah, Marcio Nucci, Caroline Besson, Federico Itri, Ozren Jaksic, Natasha Čolović, Barbora Weinbergerová, Guldane Cengiz Seval, Tatjana Adžić-Vukičević, Tomáš Szotkowski, Uluhan Sili, Michelina Dargenio, Jens van Praet, Jaap van Doesum, Martin Schönlein, Zdeněk Ráčil, Pavel Žák, Christian Bjørn Poulsen, Gabriele Magliano, Moraima Jiménez, Valentina Bonuomo, Klára Piukovics, Giulia Dragonetti, Fatih Demirkan, Ola Blennow, Toni Valković, Maria Gomes Da Silva, Johan Maertens, Andreas Glenthøj, Noemí Fernández, Rui Bergantim, Luisa Verga, Verena Petzer, Ali S. Omrani, Gustavo-Adolfo Méndez, Marina Machado, Marie-Pierre Ledoux, Rebeca Bailén, Rafael F. Duarte, Maria Ilaria Del Principe, Francesca Farina, Sonia Martín-Pérez, Julio Dávila-Valls, Monia Marchetti, Yavuz M. Bilgin, Nicola S. Fracchiolla, Chiara Cattaneo, Ildefonso Espigado, Raul Cordoba, Graham P. Collins, Jorge Labrador, Iker Falces-Romero, Lucia Prezioso, Stef Meers, Francesco Passamonti, Caterina Buquicchio, Alberto López-García, Austin Kulasekararaj, Irati Ormazabal-Vélez, Annarosa Cuccaro, Carolina Garcia-Vidal, Alessandro Busca, Milan Navrátil, Nick de Jonge, Monika M. Biernat, Anna Guidetti, Ghaith Abu-Zeinah, Michail Samarkos, Amalia Anastasopoulou, Cristina de Ramón, Tomás José González-López, Martin Hoenigl, Olimpia Finizio, László Imre Pinczés, Natasha Ali, Antonio Vena, Carlo Tascini, Zlate Stojanoski, Maria Merelli, Ziad Emarah, Milena Kohn, Aleksandra Barać, Miloš Mladenović, Bojana Mišković, Osman Ilhan, Gökçe Melis Çolak, Martin Čerňan, Stefanie K. Gräfe, Emanuele Ammatuna, Michaela Hanakova, Benjamín Víšek, Alba Cabirta, Anna Nordlander, Raquel Nunes Rodrigues, Ditte Stampe Hersby, Giovanni Paolo Maria Zambrotta, Dominik Wolf, Lucía Núñez-Martín-Buitrago, Elena Arellano, Tommaso Francesco Aiello, Ramón García-Sanz, Juergen Prattes, Matthias Egger, Alessandro Limongelli, Martina Bavastro, Milche Cvetanoski, Miriam Dibos, Sebastian Rasch, Laman Rahimli, Oliver A. Cornely, Livio Pagano, the EPICOVIDEHA registry, Joseph Meletiadis, Florian Reizine, Jan Novák, Summiya Nizamuddin, Roberta Di Blasi, Alexandra Serris, Pavel Jindra, Sylvain Lamure, François Danion, Maria Chiara Tisi, Mario Virgilio Papa, Nurettin Erben, Ľuboš Drgoňa, Nathan C. Bahr, Murtadha Al-Khabori, Ayten Shirinova, Jörg Schubert, Lisset Lorenzo De La Peña, José-Ángel Hernández-Rivas, Elena Busch, Josip Batinić, Giuseppe Sapienza, Mohammad Reza Salehi, Reham Abdelaziz Khedr, Nina Khanna, Baerbel Hoell-Neugebauer, Ana Groh, Eleni Gavriilaki, Rita Fazzi, Rémy Duléry, Roberta Della Pepa, Mario Delia, Nicola Coppola, Maria Calbacho, Darko Antić, Hossein Zarrinfer, Ayel Yahia, Vivien Wai-Man, Ana Torres-Tienza, Alina Daniela Tanasa, Andrés Soto-Silva, Laura Serrano, Enrico Schalk, Ikhwan Rinaldi, Gaëtan Plantefeve, Monica Piedimonte, Maria Enza Mitra, Carolina Miranda-Castillo, Jorge Loureiro-Amigo, Ira Lacej, Martin Kolditz, María-Josefa Jiménez-Lorenzo, Guillemette Fouquet, Omar-Francisco Coronel-Ayala, Mathias Brehon, Panagiotis Tsirigotis, Anastasia Antoniadou, Gina Varricchio, Maria Vehreschild, Agostino Tafuri, José-María Ribera-Santa Susana, Joyce Marques De Almeida, María Fernández-Galán, Avinash Aujayeb, Athanasios Tragiannidis, Malgorzata Mikulska, Sein Win, Elizabeth De Kort, Hans-Beier Ommen, Donald C. Vinh, Hans Martin Orth, Sandra Malak, Przemyslaw Zdziarski, Modar Saleh, Chi Shan Kho, Fabio Guolo, M. Mansour Ceesay, Christopher H. Heath, Sergey Gerasymchuk, Monica Fung, Maximilian Desole, Erik De Cabo, Tania Cushion, Fazle Rabbi Chowdhury, Louis Yi Ann Chai, Fevzi Altuntaş, Charlotte Flasshove

**Affiliations:** 1 Medizinische Klinik II, Klinikum rechts der Isar, TU München, Munich, Germany; 2 Faculty of Medicine, and University Hospital Cologne, Institute of Translational Research, Cologne Excellence Cluster on Cellular Stress Responses in Aging-Associated Diseases (CECAD), University of Cologne, Herderstraße 52-54, 50931 Cologne, Germany; 3 Faculty of Medicine, University Hospital Cologne, Department I of Internal Medicine, Center for Integrated Oncology Aachen Bonn Cologne Duesseldorf (CIO ABCD) and Excellence Center for Medical Mycology (ECMM), University of Cologne, Cologne, Germany; 4 German Centre for Infection Research (DZIF), Partner Site Bonn-Cologne, Cologne, Germany; 5 Hematology and Stem Cell Transplant Unit, IRCCS Regina Elena National Cancer Institute, Rome, Italy; 6 Oncology Center, Mansoura University, Mansoura, Egypt; 7 Federal University of Rio de Janeiro, Rio de Janeiro, Brazil; 8 Centre Hospitalier de Versailles, Le Chesnay, France; Université Paris-Saclay, UVSQ, Inserm, Équipe “Exposome et Hérédité”, CESP, Villejuif, France; 9 San Luigi Gonzaga Hospital - Orbassano, Orbassano, Italy; 10 Department of Hematology, University Hospital Dubrava, Zagreb, Croatia; 11 University Clinical Center Serbia, Medical Faculty University Belgrade, Belgrade, Serbia; 12 Department of Internal Medicine - Hematology and Oncology, Masaryk University Hospital Brno, Brno, Czech Republic; 13 Ankara University, Ankara, Turkey; 14 COVID hospital “Batajnica”, Belgrade, Serbia; 15 University Hospital Olomouc, Olomouc, Czech Republic; 16 Department of Infectious Diseases and Clinical Microbiology, School of Medicine, Marmara University, Istanbul, Turkey; 17 Hematology and Stem Cell Transplan Unit, Vito Fazzi Hospital, Lecce, Italy; 18 Department of Nephrology and Infectious Diseases, AZ Sint-Jan Brugge-Oostende AV, Brugge, Belgium; 19 University Medical Center Groningen, Groningen, Netherlands; 20 Department of Oncology, Hematology and Bone Marrow Transplantation with Section of Pneumology, University Medical Center Hamburg-Eppendorf, Hamburg, Germany; 21 Institute of Hematology and Blood Transfusion, Prague, Czech Republic; 22 University Hospital Hradec Králové, Hradec Králové, Czech Republic; 23 Zealand University Hospital, Roskilde, Roskilde, Denmark; 24 ASST Grande Ospedale Metropolitano Niguarda, Milan, Italy; 25 Department of Hematology, Vall d’Hebron Hospital Universitari, Experimental Hematology, Vall d’Hebron Institute of Oncology (VHIO), Vall d’Hebron Barcelona Hospital Campus, Barcelona, Spain; 26 Departament de Medicina, Universitat Autònoma de Barcelona, Bellaterra, Spain; 27 Department of Medicine, Section of Hematology, University of Verona, Verona, Italy; 28 Department of Clinical and Biological Sciences, University of Turin, Turin, Italy; 29 Department of Internal Medicine, South Division Faculty of Medicine, University of Szeged, Szeged, Hungary; 30 Hematology Unit, Fondazione Policlinico Universitario Agostino Gemelli - IRCCS, Rome, Italy; 31 Division of Hematology, Dokuz Eylul University, Izmir, Turkey; 32 Department of Infectious Diseases, Karolinska University Hospital, Stockholm, Sweden; 33 University Hospital Centre Rijeka, Rijeka, Croatia; 34 Croatian Cooperative Group for Hematological Diseases (CROHEM), Faculty of Medicine and Faculty of Health Studies, University of Rijeka, Rijeka, Croatia; 35 Portuguese Institute of Oncology, Lisbon, Portugal; 36 Department of Microbiology, Immunology, and Transplantation, KULeuven, Leuven and Department of Hematology, UZ Leuven, Louvain, Belgium; 37 Department of Hematology, Copenhagen University Hospital - Rigshospitalet, Copenhagen, Denmark; 38 Hospital Universitario Marqués de Valdecilla, Santander, Spain; 39 Centro Hospitalar e Universitário São João, Porto, Portugal; 40 Azienda Ospedaliera San Gerardo - Monza, Monza, Italy; 41 Università Milano-Bicocca, Milan, Italy; 42 Department of Hematology and Oncology, Comprehensive Cancer Center Innsbruck (CCCI), Medical University of Innsbruck (MUI), Innsbruck, Austria; 43 Communicable Disease Center, Hamad Medical Corporation, Doha, Qatar; 44 Hospital Escuela de Agudos Dr. Ramón Madariaga, Posadas, Argentina; 45 Clinical Microbiology and Infectious Diseases Department, Hospital General Universitario Gregorio Marañón, Madrid, Spain; 46 ICANS, Strasbourg, France; 47 Hematology Department, Hospital General Universitario Gregorio Marañón, Madrid, Spain; 48 Hospital Universitario Puerta de Hierro, Majadahonda, Spain; 49 Hematology, Department of Biomedicine and Prevention, University of Rome Tor Vergata, Rome, Italy; 50 IRCCS Ospedale San Raffaele, Milan, Italy; 51 Hospital Nuestra Señora de Sonsoles, Ávila, Spain; 52 Azienda Ospedaliera Nazionale SS. Antonio e Biagio e Cesare Arrigo, Alessandria, Italy; 53 Department of Internal Medicine, ADRZ, Goes, Netherlands; 54 Fondazione IRCCS Ca’ Granda Ospedale Maggiore Policlinico, Milan, Italy; 55 Hematology Unit, ASST-Spedali Civili, Brescia, Italy; 56 Department of Hematology, University Hospital Virgen Macarena - University Hospital Virgen del Rocío, Instituto de Biomedicina de Sevilla (IBIS / CSIC), Universidad de Sevilla (Departamento de Medicina), Seville, Spain; 57 Fundacion Jimenez Diaz University Hospital, Health Research Institute IIS-FJD, Madrid, Spain; 58 NIHR Oxford Biomedical Research Centre, Churchill Hospital, Oxford, UK; 59 Department of Hematology, Research Unit, Hospital Universitario de Burgos, Burgos, Spain; 60 Facultad de Ciencias de la Salud, Universidad Isabel I, Burgos, Spain; 61 La Paz University Hospital, Madrid, Spain; 62 CIBERINFEC, Instituto de Salud Carlos III, Madrid, Spain; 63 Hospital University of Parma - Hematology and Bone Marrow Unit, Parma, Italy; 64 AZ KLINA, Brasschaat, Belgium; 65 Department of Medicine and Surgery, University of Insubria and ASST Sette Laghi, Ospedale di Circolo of Varese, Varese, Italy; 66 Ematologia con Trapianto, Ospedale Dimiccoli Barletta, Barletta, Italy; 67 King’s College Hospital, London, UK; 68 King’s College London, London, UK; 69 Complejo Hospitalario de Navarra, Iruña-Pamplona, Spain; 70 Hematology Unit, Center for Translational Medicine, Azienda USL Toscana NordOvest, Leghorn, Italy; 71 Hospital Clinic, Barcelona, Spain; 72 Stem Cell Transplant Center, AOU Citta’ della Salute e della Scienza, Turin, Italy; 73 Head of the ICU and Transplant Unit, Department of Hematooncology, University Hospital of Ostrava, Ostrava-Poruba, Czech Republic; 74 Amsterdam UMC, location VUmc, Amsterdam, Netherlands; 75 Department of Haematology, Blood Neoplasms, and Bone Marrow Transplantation, Wroclaw Medical University, Wroclaw, Poland; 76 University of Milan and Fondazione IRCCS Istituto Nazionale Dei Tumori, Milan, Italy; 77 Division of Hematology and Oncology, Weill Cornell Medicine, New York, USA; 78 Laikon Hospital, Athens, Greece; 79 Hematology Department, Hospital Universitario de Salamanca, Salamanca, Spain; 80 IBSAL, Centro de Investigación del Cáncer-IBMCC (USAL-CSIC), Salamanca, Spain; 81 Department of Hematology, Hospital Universitario de Burgos, Burgos, Spain; 82 Division of Infectious Diseases, Department of Internal Medicine, Medical University of Graz, Graz, Austria; 83 BioTechMed, Graz, Austria; 84 UOC Hematology, AORN Cardarelli, Naples, Italy; 85 Division of Hematology, Department of Internal Medicine, Faculty of Medicine, University of Debrecen, Debrecen, Hungary; 86 Aga Khan University, Karachi, Pakistan; 87 Ospedale Policlinico San Martino, Genoa, Italy; 88 Azienda Sanitaria Universitaria del Friuli Centrale, Udine, Italy; 89 University Clinic of Hematology, Skopje, North Macedonia; 90 Centre Hospitalier de Versailles, Versailles, France; 91 Clinic for Infectious and Tropical Diseases, University Clinical Center of Serbia, Faculty of Medicine, University of Belgrade, Belgrade, Serbia; 92 COVID hospital ““Batajnica””, Belgrade, Serbia; 93 Clinic for Orthopedic Surgery and Traumatology, University Clinical Center of Serbia, Belgrade, Serbia; 94 Center for Radiology, University Clinical Center of Serbia, Belgrade, Serbia; 95 Department of Hemato-Oncology, Faculty of Medicine and Dentistry, Palacky University and University Hospital Olomouc, Olomouc, Czech Republic; 96 Department of Medicine, University Medical Center Hamburg-Eppendorf, Hamburg, Germany; 97 Department of Hematology and Oncology, Medical University of Innsbruck, Innsbruck, Austria; 98 Head of Molecular Biology an HLA Unit, Department of Hematology, University Hospital of Salamanca (HUS/IBSAL/CIBERONC), Salamanca, Spain; 99 Medical University of Graz, Graz, Austria; 100 Faculty of Medicine, and University Hospital Cologne, Clinical Trials Centre Cologne (ZKS Köln), University of Cologne, Cologne, Germany; 101 Faculty of Medicine, and University Hospital Cologne, Center for Molecular Medicine Cologne (CMMC), University of Cologne, Cologne, Germany; 102 Hematology Unit, Università Cattolica del Sacro Cuore, Rome, Italy

## Introduction

The risk for a severe coronavirus disease 2019 (COVID-19) with need for an intensive care unit (ICU) admission in a non-immunocompromised vaccinated population dropped from 5% at the beginning of the pandemic to at least 0.2% and is still decreasing since the omicron strain dominates the COVID-19 pandemic [1]. Beyond the risk factors identified for a severe acute respiratory syndrome coronavirus 2 (SARS-CoV-2) infection like male sex, older age, and comorbidities such as cardiovascular disease, lung disease or obesity, patients with a history of malignancy, specifically patients with haematological malignancy, are prone to develop a complicated SARS-CoV-2 infection with need for ICU which is still associated with poorer clinical outcome [2–15]. The circumstances of a widely heterogenous population with regards to the type of haematological malignancy, extent of disease, haematological malignancy treatment history, [16–18] and baseline performance status are even more challenging in the environment of an ICU [19]. Although, data referring to critically ill COVID-19 patients regarding treatment strategies and outcome are widely available, data referring to critically ill patients with haematological malignancy are scarce and underreported [20].

The aim of this study is to analyze the epidemiology, risk factors and outcome of patients with haematological malignancy with need for an ICU setting using the data from the large-scale EPICOVIDEHA registry of the European Hematology Association—Scientific Working Group Infectious in Hematology (EHA-SWG) [21].

## Methods

### Study design, patients, and procedures

From January 1st, 2021, until March 10th, 2022, participating institutions documented retrospectively episodes of COVID-19 in their patients with haematological malignancy. Our analysis comprised data from the EPICOVIDEHA registry [21]. EPICOVIDEHA (www.clinicaltrials.gov; National Clinical Trials identifier NCT04733729) is an international open web-based registry for patients with haematological malignancy infected with SARS-CoV-2. EPICOVIDEHA was approved by the local ethics committee of the Fondazione Policlinico Universitario Agostino Gemelli—IRCCS, Università Cattolica del Sacro Cuore of Rome, Italy (Study ID: 3226). When applicable, the respective local ethics committee of each participating institution have approved the project. EPICOVIDEHA methods have been described elsewhere [21]. The electronic case report form (eCRF) is accessible online at www.clinicalsurveys.net (EFS Summer 2021, TIVIAN, Cologne, Germany). Each documented patient was reviewed and validated by infectious diseases and haematology experts from the coordination team. Inclusion criteria were (a) active malignancies within the last 5 years before COVID-19 diagnosis, or day 0, (b) patients ≥ 18 years old, (c) laboratory-based diagnosis of SARS-CoV-2 infection, and (d) last vaccine dose 15 or more days before PCR confirmed SARS-CoV-2 infection. Data on baseline conditions pre-COVID-19 (i.e., age, sex, status of haematological malignancy at COVID-19 diagnosis, defined as active [onset and refractory/resistant], stable disease or controlled [complete and partial response] based on the reports from the respective participating institution, factors predisposing for COVID-19), clinical management of the haematological malignancy (i.e., last treatment strategy), vaccine type, spike protein concentration at diagnosis of COVID-19, COVID-19 diagnosis and management (i.e., reason for diagnostic test, symptoms at onset, hospital stay/admission during infection, if needed, treatments received for infection) and outcome (i.e., mortality, attributable cause of death, mortality [assessed by the medical team in charge of the patient], last day of follow-up) were collected.

### Study objectives

This study aimed to achieve several key objectives related to patients with haematological malignancies and COVID-19 who required ICU admission. Firstly, we wanted to comprehensive describe the sample of patients registered in EPICOVIDEHA who needed ICU care. Secondly, we intended to conduct a thorough analysis to identify factors associated with ICU admission, seeking to uncover potential predictors that could contribute to patients being eventually admitted to an ICU unit. Thirdly, we ambitioned to compare mortality rates between patients admitted to the ICU and those managed outside the ICU, offering valuable insights into the outcomes of these distinct patient groups. Lastly, we endeavoured to perform an in-depth analysis of the factors associated with mortality after ICU admission, shedding light on the determinants of survival for patients facing this critical phase of their illness.

### Sample size and statistical analysis

No a priori sample size calculation was performed for this analysis. Categorical variables are presented with frequencies and percentages, and continuous variables with median, interquartile range (IQR) and absolute range. Proportion comparisons were performed using Fisher's exact or Pearson's chi (X) squared tests, respectively.

Logistic regression was utilized to determine which independent variables were associated with subsequent ICU admission. Additionally, Cox regression was used to analyze which factor could be associated with mortality 365 days after COVID-19 diagnosis in ICU patients who had data on duration of follow-up. Variables with a *p*-value < 0.1 in the univariable models were considered for the respective multivariable model. Clinical significance of the respective variable was also considered, based on previously reported literature, before transfer to multivariable analysis. A log-rank test was used to compare the survival probability of the patients admitted in the ICU based adjusted by anti-SARS-CoV-2 vaccination prior to COVID-19 diagnosis, which was graphically represented with a Kaplan–Meier survival plot. Patients with missing data in essential fields (i.e., haematological malignancies, chemotherapeutic program, vaccination status, COVID-19 management, or survival status) were considered as not valid and then, excluded from the final analysis. Among the valid cases, if a value in a specific variable was missing or unknown, it is indicated as such in the descriptive analysis. Patients with missing data in a certain variable were excluded from regression analyses in case that variable was included into such analyses.

A *p*-value ≤ 0.05 was considered statistically significant. SPSSv25.0 was employed for statistical analyses (SPSS, IBM Corp., Chicago, IL, United States).

## Results

### Cohort characteristics

A total of 94 centres in 26 countries, mainly from Europe, participated and registered 6934 cases. The clinical characteristics of these evaluable cases are reported in Table 1. Lymphoid malignancies were the largest subgroup, accounting 2414 cases (34.8%); the most frequently reported diagnosis was non-Hodgkin lymphoma (NHL, 2137 cases, 30.8%). Among myeloid malignancies, the most frequent diagnosis was acute myeloid leukaemia (AML, 828 cases, 11.9%). At the time of COVID-19 diagnosis, most patients had a controlled haematological malignancy (*n* = 3257, 47%), or a stable disease (*n* = 1305, 18.8%) and the remaining 31% (*n* = 2151 cases) an active disease. The most frequently reported last haematological malignancy treatment within the last 3 months was immuno-chemotherapy (*n* = 4367, 63.0%), and 241 patients with haematological malignancy (3.5%) had received HSCT within 6 months before COVID-19 diagnosis (allogeneic: 145; autologous: 96) and 28 had chimeric antigen receptor T cells (CAR-T) therapy (see Table 1).

**Table 1:** Baseline characteristics, treatments, and outcome of patients with haematological malignancy and COVID-19, by need of intensive care

	Overall		No ICU		ICU		*p*-value
	*n*	%	*n*	%	*n*	%	
Sex							
Female	2873	41.4%	2463	42.1%	410	38.0%	**0.012**
Male	4061	58.6%	3391	57.9%	670	62.0%	
Age	65 (54–75) [18–97]		65 (53–75) [18–97]		65 (55–73) [18–92]		0.057
18–25 years old	214	3.1%	184	3.1%	30	2.8%	** < 0.001**
26–50 years old	1198	17.3%	1044	17.8%	154	14.3%	
51–69 years old	2784	40.1%	2275	38.9%	509	47.1%	
> 69 years old	2738	39.5%	2351	40.2%	387	35.8%	
Comorbidities at COVID-19 diagnosis							
No comorbidities	2691	38.8%	2336	39.9%	355	32.9%	** < 0.001**
1 comorbidity	2179	31.4%	1831	31.3%	348	32.2%	
2 comorbidities	1233	17.8%	1007	17.2%	226	20.9%	
3 or more comorbidities	831	12.0%	680	11.6%	151	14.0%	
Chronic cardiopathy	2333	33.6%	1948	33.3%	385	35.6%	0.130
Chronic pulmonary disease	973	14.0%	779	13.3%	194	18.0%	** < 0.001**
Diabetes mellitus	1020	14.7%	827	14.1%	193	17.9%	**0.001**
Liver disease	285	4.1%	227	3.9%	58	5.4%	**0.023**
Obesity	562	8.1%	441	7.5%	121	11.2%	** < 0.001**
Renal impairment	509	7.3%	411	7.0%	98	9.1%	**0.017**
Smoking history	855	12.3%	711	12.1%	144	13.3%	0.275
Leukocytes	5080 (2970–8300) [4–658000]		5070 (3000–8100) [1–399000]		5000 (2400–9640) [7–658000]		0.764
Neutrophils	3000 (1660–5200) [1–391000]		3000 (1690–5080) [1–391000]		3200 (1440–5900) [1–116000]		0.108
< 501	489	7.1%	381	6.5%	108	10.0%	**0.002**
501–999	381	5.5%	311	5.3%	70	6.5%	
> 999	4950	71.4%	4157	71.0%	793	73.4%	
Lymphocytes	900 (420–1730) [1–583300]		950 (470–1800) [0.7–363000]		610 (300–1500) [2–583300]		** < 0.001**
< 201	642	9.3%	463	7.9%	179	16.6%	** < 0.001**
201–499	988	14.2%	782	13.4%	206	19.1%	
> 499	4217	60.8%	3623	61.9%	594	55.0%	
Baseline haematological malignancy							
Leukaemia	2826	40.8%	2348	40.1%	478	44.3%	0.068
Acute lymphoid leukaemia	310	4.5%	259	4.4%	51	4.7%	
Chronic lymphoid leukaemia	915	13.2%	740	12.6%	175	16.2%	
Acute myeloid leukaemia	828	11.9%	670	11.4%	158	14.6%	
Chronic myeloid leukaemia	259	3.7%	237	4.0%	22	2.0%	
Myelodysplastic syndrome	464	6.7%	409	7.0%	55	5.1%	
Hairy cell leukaemia	50	0.7%	33	0.6%	17	1.6%	
Lymphoma	2414	34.8%	2041	34.9%	373	34.5%	
Hodgkin lymphoma	277	4.0%	253	4.3%	24	2.2%	
Non-Hodgkin lymphoma	2137	30.8%	1788	30.5%	349	32.3%	
PH negative myeloproliferative diseases	450	6.5%	393	6.7%	57	5.3%	
Essential thrombocythemia	120	1.7%	112	1.9%	8	0.7%	
Myelofibrosis	195	2.8%	161	2.8%	34	3.1%	
Polycythaemia vera	113	1.6%	99	1.7%	14	1.3%	
Systemic mastocytosis	22	0.3%	21	0.4%	1	0.1%	
Plasma cell disorders	1195	17.2%	1027	17.5%	168	15.6%	
Multiple myeloma	1172	16.9%	1005	17.2%	167	15.5%	
Amyloid light-chain amyloidosis	23	0.3%	22	0.4%	1	0.1%	
Aplastic anaemia	49	0.7%	45	0.8%	4	0.4%	
Haematological malignancy status at COVID-19 diagnosis							
Controlled disease	3257	47.0%	2803	47.9%	454	42.0%	**0.001**
Stable disease	1305	18.8%	1164	19.9%	141	13.1%	
Active disease	2151	31.0%	1733	29.6%	418	38.7%	
Unknown	221	3.2%	154	2.6%	67	6.2%	
Last haematological malignancy treatment immediately before COVID-19 diagnosis							
alloHSCT							
In the last 6 months	145	2.1%	123	2.1%	22	2.0%	
> 6 months	212	3.1%	186	3.2%	26	2.4%	
autoHSCT							
In the last 6 months	96	1.4%	82	1.4%	14	1.3%	
> 6 months	59	0.9%	52	0.9%	7	0.6%	
Not reported	1	0.0%	1	0.0%	0	0.0%	
CAR-T							
In the last 6 months	28	0.4%	19	0.3%	9	0.8%	
> 6 months	29	0.4%	23	0.4%	6	0.6%	
Chemotherapy							
In the last month	3614	52.1%	3034	51.8%	580	53.7%	
In the last 3 months	753	10.9%	631	10.8%	122	11.3%	
> 3 months	794	11.5%	679	11.6%	115	10.6%	
Not reported	127	1.8%	104	1.8%	23	2.1%	
No treatment	923	13.3%	776	13.3%	147	13.6%	
Supportive therapy							
> 3 months	1	0.0%	1	0.0%	0	0.0%	
Not reported	140	2.0%	132	2.3%	8	0.7%	
Unknown	12	0.2%	11	0.2%	1	0.1%	
SARS-CoV-2 vaccine doses before COVID-19 diagnosis							
Not vaccinated	4857	70.0%	3983	68.0%	874	80.9%	** < 0.001**
One dose	182	2.6%	159	2.7%	23	2.1%	
Two doses	889	12.8%	787	13.4%	102	9.4%	
Three doses	938	13.5%	859	14.7%	79	7.3%	
Four doses	68	1.0%	66	1.1%	2	0.2%	
Type last SARS-CoV-2 vaccine before COVID-19 diagnosis							
mRNA							
BioNTech/Pfizer	1490	21.5%	1345	23.0%	145	13.4%	
Moderna COVE	364	5.2%	335	5.7%	29	2.7%	
Vector-based							
AstraZeneca Oxford	107	1.5%	95	1.6%	12	1.1%	
Sputnik	15	0.2%	14	0.2%	1	0.1%	
J&J—Janssen	25	0.4%	23	0.4%	2	0.2%	
Inactivated							
CoronaVac | Sinovac	23	0.3%	18	0.3%	5	0.5%	
Sinopharm	44	0.6%	33	0.6%	11	1.0%	
COVID-19 wave							
WT wave	3796	54.7%	3122	53.3%	674	62.4%	**0.001**
Alpha/Beta/Gamma wave	524	7.6%	410	7.0%	114	10.6%	
Delta wave	694	10.0%	563	9.6%	131	12.1%	
Omicron wave	1920	27.7%	1759	30.0%	161	14.9%	
Variant of concern							
Wild type	222	3.2%	157	2.7%	65	6.0%	** < 0.001**
Alpha	79	1.1%	57	1.0%	22	2.0%	
Beta	2	0.0%	1	0.0%	1	0.1%	
Delta	205	3.0%	169	2.9%	36	3.3%	
Omicron	796	11.5%	733	12.5%	63	5.8%	
Not tested	5630	81.2%	4737	80.9%	893	82.7%	
COVID-19 severity							
Asymptomatic	1295	18.7%	1295	22.1%	0	0.0%	**0.001**
Mild infection	1934	27.9%	1934	33.0%	0	0.0%	
Severe infection	2625	37.9%	2625	44.8%	0	0.0%	
Critical infection	1080	15.6%	0	0.0%	1080	100.0%	
COVID-19 onset symptoms							
Pulmonary	2415	34.8%	1944	33.2%	471	43.6%	**0.001**
Pulmonary + extrapulmonary	1727	24.9%	1340	22.9%	387	35.8%	
Extrapulmonary	1303	18.8%	1181	20.2%	122	11.3%	
Screening	1489	21.5%	1389	23.7%	100	9.3%	
COVID-19 treatment							
No specific treatment reported	1523	22.0%	1417	24.2%	106	9.8%	**0.001**
Antivirals +/− corticosteroids +/− plasma	700	10.1%	553	9.4%	147	13.6%	
Antivirals + monoclonal antibodies +/− corticosteroids +/− plasma	209	3.0%	173	3.0%	36	3.3%	
Monoclonal antibodies +/− corticosteroids +/− plasma	527	7.6%	484	8.3%	43	4.0%	
Plasma +/− corticosteroids	74	1.1%	52	0.9%	22	2.0%	
Corticosteroids	621	9.0%	444	7.6%	177	16.4%	
Unknown	3280	47.3%	2731	46.7%	549	50.8%	
Stay during COVID-19 episode							
Home	2328	33.6%	2328	39.8%	0	0.0%	**0.001**
Hospital	4605	66.4%	3525	60.2%	1080	100.0%	
Length of stay	14 (7–25) [1–235]		12 (7–21) [1–200]		22 (12–37) [1–235]		**0.001**
ICU	1080	15.6%					
Length of stay	10 (5–19) [1–115]		(–) [–]		10 (5–19) [1–115]		
Non-invasive ventilation	533	7.7%	0	0.0%	533	49.4%	
Invasive ventilation	421	6.1%	0	0.0%	421	39.0%	
Outcome							
Observation time, days	52 (18–155) [0–792]		61 (20–162) [0–792]		30 (14–91) [0–763]		**0.001**
Observation time, days alive	85 (28–200) [0–792]		82 (27–192.5) [0–792]		125 (42–270) [1–665]		** < 0.001**
Observation time, days dead	18 (8–38) [0–763]		16 (7–44.5) [0–657]		19 (10–33) [0–763]		0.214
Alive, d365	5200	75.0%	4792	81.9%	408	37.8%	**0.001**
Dead, d365	1734	25.0%	1062	18.1%	672	62.2%	

Bold indicates statistically significant difference

A comparison of proportions was performed for categorical data and a median difference analysis for continuous variables

### Need for ICU

A total of 3705 (53.5%) patients with haematological malignancy developed severe COVID-19 involving need for hospitalization. Out of these, 1080 patients with haematological malignancy (29.2%, 15.6% from the whole cohort) developed a critical infection, and thus needed for ICU care. Invasive mechanical ventilation was needed in 421 (39%), while 533 (49.4%) received non-invasive ventilation support. The median duration of ICU stay was 10 days (IQR 5–19; range 1–115). The overall hospital stay was significantly longer (*p* < 0.001) in the ICU group as compared to the non-ICU group (22 vs. 12 days; (IQR 12–37, range 1–235 vs. IQR 7–21, range 1–200, *p* = 0.001).

### Factors associated with critical COVID-19 infection

#### General factors and co-morbidities

groupMale sex was associated with critical infection (*p* = 0.033, OR 1.176, 95% CI 1.013–1.365). Most patients with haematological malignancy presented at least one comorbidity (*n* = 4243, 61.2% of the total cohort). This proportion was significantly higher in the ICU group as compared to the non-ICU group (67.1% vs. 60.1%, *p* < 0.001). Chronic cardiopathy (*n* = 385; 35.6%), chronic pulmonary disease (*n* = 194; 18%) and diabetes (*n* = 193; 17.9%) were the leading comorbidities in the ICU group. Also pulmonary in combination with extrapulmonary symptoms at COVID-19 onset (*p* = 0.023, OR 1.216, 95% CI 1.028–1.440) or extrapulmonary syndromes alone at COVID-19 onset (*p* < 0.001, OR 0.464, 95% CI 0.367–0.586) were associated with critically illness and need for intensive care. A complete list of comorbidities and the results of the univariate and multivariate analysis are available in Table 1, 2 and 3.

**Table 2:** Logistic regression of variables associated with ICU admission

	Univariable				Multivariable			
	*p*-value	OR	95% CI		*p*-value	OR	95% CI	
			Lower	Upper			Lower	Upper
Age	0.403	0.998	0.994	1.002				
Sex	**0.012**	1.187	1.039	1.356	**0.033**	1.176	1.013	1.365
Comorbidities at COVID-19 diagnosis								
No comorbidities	–	–	–	–				
1 comorbidity	**0.006**	1.251	1.066	1.468				
2 comorbidities	**0.000**	1.477	1.231	1.772				
3 or more comorbidities	**0.000**	1.461	1.186	1.800				
Chronic cardiopathy	0.130	1.111	0.970	1.272				
Chronic pulmonary disease	**0.000**	1.426	1.200	1.695	0.071	1.191	0.985	1.440
Diabetes mellitus	**0.001**	1.323	1.113	1.571	0.408	1.089	0.890	1.332
Liver disease	**0.024**	1.407	1.046	1.891	0.082	1.337	0.964	1.853
Obesity	**0.000**	1.549	1.252	1.915	** < 0.001**	1.516	1.192	1.928
Renal impairment	**0.018**	1.322	1.049	1.664	0.593	1.075	0.824	1.402
Smoking history	0.276	1.113	0.918	1.349				
No risk factor identified	**0.001**	0.731	0.637	0.839	0.206	0.895	0.755	1.063
Neutrophils								
< 501	–	–	–	–	–	–	–	–
501–999	0.190	0.799	0.572	1.117	0.848	0.965	0.673	1.384
> 999	**0.001**	0.677	0.541	0.849	0.922	1.013	0.779	1.317
Lymphocytes								
< 201	–	–	–	–	–	–	–	–
201–499	**0.001**	0.683	0.543	0.861	**0.006**	0.709	0.554	0.906
> 499	**0.001**	0.425	0.351	0.516	** < 0.001**	0.464	0.377	0.572
Baseline haematological malignancy								
Leukaemia	–	–	–	–	–	–	–	–
Lymphoma	0.845	1.015	0.876	1.176	0.300	0.914	0.772	1.083
PH negative myeloproliferative diseases	0.060	0.753	0.560	1.012	0.888	1.024	0.733	1.431
Plasma cell disorders	0.095	0.849	0.701	1.029	0.063	0.819	0.663	1.011
Aplastic anaemia	0.140	0.462	0.165	1.290	0.260	0.545	0.189	1.568
Haematological malignancy status at COVID-19 diagnosis								
Controlled disease	–	–	–	–	–	–	–	–
Stable disease	**0.005**	0.748	0.612	0.914	**0.003**	0.710	0.567	0.888
Active disease	** < 0.001**	1.489	1.287	1.723	** < 0.001**	1.354	1.150	1.593
Unknown	** < 0.001**	2.686	1.983	3.639	** < 0.001**	2.165	1.546	3.033
SARS-CoV-2 vaccine doses before COVID-19 diagnosis								
Not vaccinated	–	–	–	–	–	–	–	–
One dose	0.065	0.659	0.423	1.027	0.172	0.697	0.415	1.169
Two doses	** < 0.001**	0.591	0.475	0.735	**0.005**	0.693	0.535	0.898
Three doses	** < 0.001**	0.419	0.329	0.534	** < 0.001**	0.607	0.456	0.807
Four doses	**0.006**	0.138	0.034	0.565	**0.026**	0.198	0.047	0.827
Type last SARS-CoV-2 vaccine before COVID-19 diagnosis								
mRNA	–	–	–	–				
Vector-based	0.744	1.097	0.629	1.914				
Inactivated	** < 0.001**	3.029	1.691	5.426				
COVID-19 wave								
WT wave	–	–	–	–				
Alpha/Beta/Gamma wave	**0.027**	1.288	1.030	1.611				
Delta wave	0.479	1.078	0.876	1.326				
Omicron wave	** < 0.001**	0.424	0.354	0.508				
Variant of concern								
Wild type	–	–	–	–	–	–	–	–
Alpha	0.810	0.932	0.527	1.649	0.895	0.958	0.510	1.800
Beta	0.535	2.415	0.149	39.201	1.000			
Delta	**0.005**	0.515	0.324	0.816	0.089	0.636	0.378	1.071
Omicron	** < 0.001**	0.208	0.141	0.306	** < 0.001**	0.291	0.187	0.454
Not tested	** < 0.001**	0.455	0.338	0.613	** < 0.001**	0.516	0.372	0.715
COVID-19 onset symptoms								
Pulmonary	–	–	–	–	–	–	–	–
Pulmonary + extrapulmonary	**0.023**	1.192	1.025	1.387	0.**023**	1.216	1.028	1.440
Extrapulmonary	** < 0.001**	0.426	0.345	0.527	** < 0.001**	0.464	0.367	0.586
Screening	** < 0.001**	0.297	0.237	0.373	** < 0.001**	0.304	0.236	0.392

Bold indicates statistically significant difference

**Table 3:** Cox regression of variables associated with mortality in patients admitted to ICU

	Univariable				Multivariable			
	*p*-value	HR	95% CI		*p*-value	HR	95% CI	
			Lower	Upper			Lower	Upper
Age	**0.001**	1.010	1.004	1.016	**0.010**	1.008	1.002	1.015
Sex	0.401	0.935	0.800	1.093				
Comorbidities								
No comorbidities	–	–	–	–				
1 comorbidity	0.785	1.027	0.849	1.241				
2 comorbidities	0.589	1.061	0.857	1.313				
3 or more comorbidities	0.071	1.242	0.981	1.572				
Chronic cardiopathy	**0.006**	1.247	1.066	1.459	0.324	1.095	0.914	1.311
Chronic pulmonary disease	0.520	0.937	0.769	1.142				
Diabetes mellitus	**0.002**	1.350	1.117	1.632	**0.003**	1.366	1.114	1.675
Liver disease	**0.006**	1.549	1.137	2.110	** < 0.001**	1.782	1.282	2.476
Obesity	**0.014**	0.724	0.559	0.937	**0.020**	0.718	0.543	0.949
Renal impairment	**0.001**	1.485	1.164	1.894	**0.021**	1.354	1.048	1.750
Smoking history	0.495	1.078	0.869	1.337				
No risk factor identified	0.519	0.948	0.805	1.116				
Neutrophils								
< 501	–	–	–	–	–	–	–	–
501–999	0.350	0.846	0.595	1.202	0.780	0.951	0.666	1.357
> 999	**0.006**	0.715	0.563	0.909	**0.032**	0.766	0.600	0.977
Lymphocytes								
< 201	–	–	–	–				
201–499	0.105	0.813	0.634	1.044				
> 499	0.411	0.918	0.749	1.125				
Baseline haematological malignancy								
Leukaemia	–	–	–	–	–	–	–	–
Lymphoma	0.054	0.900	0.808	1.002	**0.036**	0.819	0.679	0.987
PH negative myeloproliferative diseases	** < 0.001**	0.689	0.553	0.857	0.873	1.033	0.695	1.534
Plasma cell disorders	0.301	0.931	0.814	1.066	0.759	1.038	0.820	1.313
Aplastic anaemia	0.220	0.663	0.344	1.279	0.293	0.472	0.116	1.915
Haematological malignancy status at COVID-19 diagnosis								
Controlled disease	–	–	–	–	–	–	–	–
Stable disease	0.983	1.003	0.773	1.300	0.237	0.843	0.635	1.119
Active disease	** < 0.001**	1.675	1.415	1.983	** < 0.001**	1.593	1.329	1.908
Unknown	** < 0.001**	2.409	1.774	3.270	** < 0.001**	2.404	1.747	3.308
SARS-CoV-2 vaccine doses before COVID-19 diagnosis								
Not vaccinated	–	–	–	–				
One dose	0.836	0.944	0.544	1.636				
Two doses	0.669	0.941	0.713	1.242				
Three doses	0.110	0.759	0.542	1.064				
Four doses	0.922	0.000	0.000					
Type last SARS-CoV-2 vaccine before COVID-19 diagnosis								
mRNA	–	–	–	–				
Vector-based	0.888	0.942	0.410	2.162				
Inactivated	**0.030**	1.957	1.067	3.588				
COVID-19 wave								
WT wave	–	–	–	–				
Alpha/Beta/Gamma wave	0.265	1.149	0.900	1.467				
Delta wave	0.080	1.232	0.975	1.557				
Omicron wave	0.979	1.003	0.787	1.279				
Variant of concern								
Wild type	–	–	–	–				
Alpha	0.732	0.893	0.468	1.704				
Beta	0.918	0.000	0.000					
Delta	0.680	0.887	0.502	1.567				
Omicron	0.489	0.840	0.512	1.377				
Not tested	0.095	1.315	0.953	1.813				
COVID-19 onset symptoms								
Pulmonary	–	–	–	–				
Pulmonary + extrapulmonary	0.991	0.999	0.841	1.186				
Extrapulmonary	0.840	1.026	0.799	1.318				
Screening	0.715	1.051	0.804	1.374				
COVID-19 treatment								
No specific treatment reported	–	–	–	–	–	–	–	–
Antivirals +/− corticosteroids +/− plasma	0.298	1.179	0.864	1.608	0.184	1.252	0.898	1.745
Antivirals + monoclonal antibodies +/− corticosteroids +/− plasma	**0.003**	0.376	0.199	0.713	**0.012**	0.422	0.214	0.830
Monoclonal antibodies +/− corticosteroids +/− plasma	0.075	0.629	0.377	1.049	0.268	0.741	0.437	1.258
Plasma +/− corticosteroids	0.216	1.400	0.821	2.388	0.262	1.389	0.782	2.465
Corticosteroids	0.831	1.034	0.761	1.405	0.219	1.230	0.884	1.710
Unknown	0.612	0.934	0.717	1.216	0.867	0.976	0.734	1.298

Bold indicates statistically significant difference

#### Viral subtypes

**Fig. 1:**
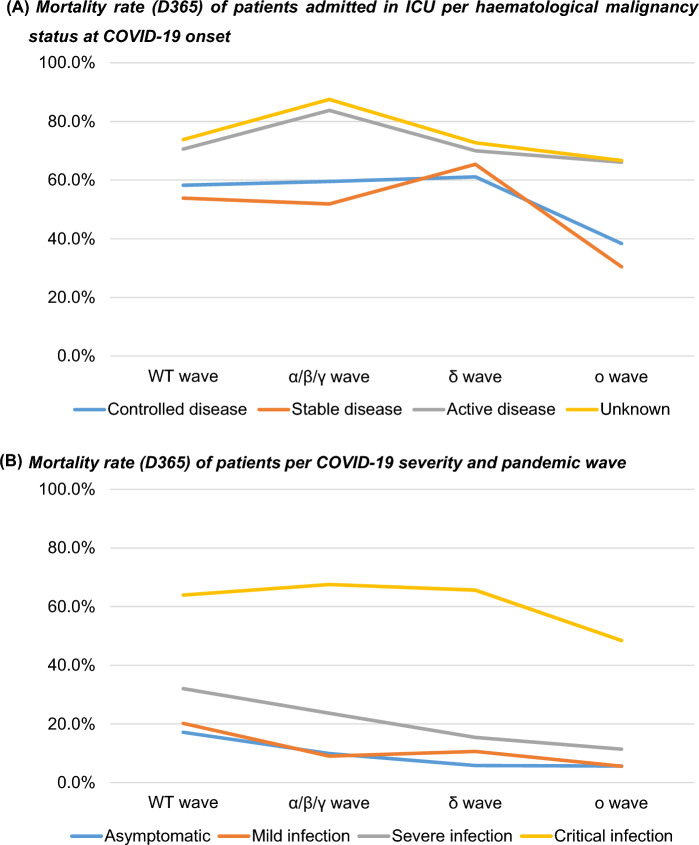
Mortality rate *(D365)* of patients (**A**) admitted in ICU per haematological malignancy status at COVID-19 onset and (**B**) per COVID-19 severity and pandemic wave

Omicron strain reduced the risk as compared to the other viral subtypes (*p* < 0.001, OR 0.291, 95% CI 0.187–0.454) for the need of ICU care in patients with haematological malignancy, which can also be seen in the proportion of critical COVID-19 infections (see Fig. 1).

#### Underlying hematological malignancy

In univariable and multivariable analyses, active malignancy (*p* < 0.001, OR 1.354, 95% CI 1.150–1.593) was associated with critically illness and need for intensive care (see also Table 1, 2, 3).

#### Vaccination

Unvaccinated patients with haematological malignancy were found more often in the ICU group (80.9% versus 68%; *p* < 0.001) and patients with haematological malignancy and at least two vaccine doses were significantly (*p* = 0.005, OR 0.693, 95% CI 0.535–0.898), three (*p* < 0.001, OR 0.607, 95% CI 0.456–0.807) or four vaccines (*p* = 0.026, OR 0.198, 95% CI 0.047–0.827) were less often transferred to the ICU.

### ICU and outcome: exploratory analysis

#### Mortality rate

**Fig. 2:**
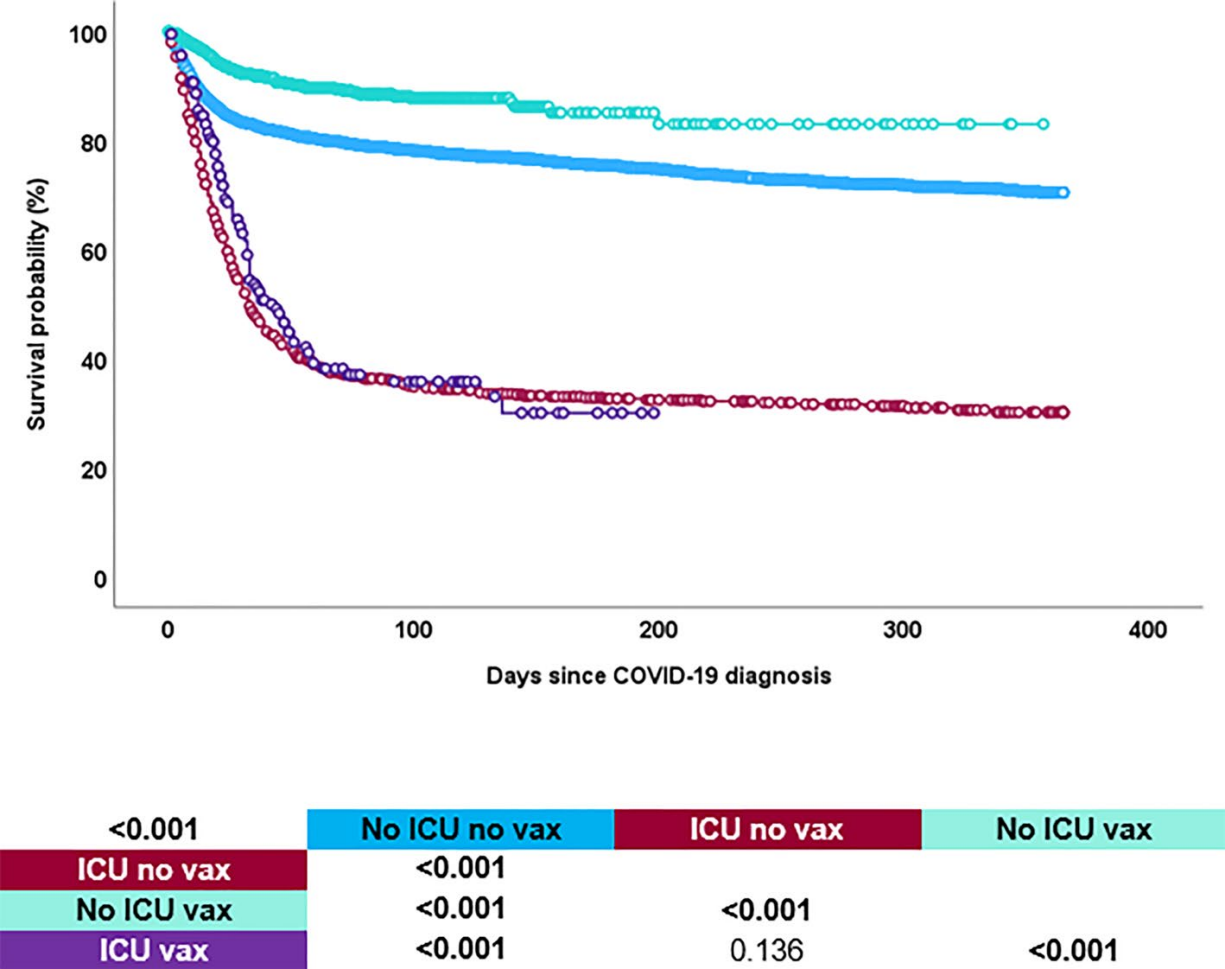
Survival probability of patients with haematological malignancy and COVID-19, by need for intensive care and previous vaccination

With 62.8% (*n* = 678) the mortality rate was significantly higher (*p* < 0.001) in the ICU group as compared to the non-ICU group (18.6%, *n* = 1086) (see Fig. 2, Supplementary Fig. S1).

#### Mortality rate associated risk factors

Using univariable and multivariable analyses we found active haematological malignancy (*p* < 0.001, HR 1.593, 95% CI 1.329–1.908), age (*p* = 0.010, HR 1.008, 95% CI 1.002–1.015) and comorbidities such as diabetes (*p* = 0.003, HR 1.366, 95% CI 1.114–1.675), liver disease (*p* < 0.001, HR 1.782, 95% CI 1.282–2.476); obesity (*p* = 0.020, HR 0.718, 95% CI 0.543–0.949) and renal impairment (*p* = 0.021, HR 1.354, 95% CI 1.048–1.750) as factors that increased ICU mortality. The influence of the SARS-CoV-2 subtypes and the effect on the mortality rate through the pandemic are presented in Fig. 1.

#### Factors associated with mortality rate reduction

COVID-19 treatment with antivirals, monoclonal antibodies, and corticosteroids (*p* = 0.012, HR 0.422, 95% CI 0.214–0.830) was associated with significantly reduced mortality rates in critically ill patients with haematological malignancy.

## Discussion

In COVID-19 patients with haematological malignancy studies on need for intensive care and outcomes are scarce. We identified factors associated with admission into intensive care units in 6934 such high-risk patients with haematological malignancy, and investigated variables associated with mortality in these ICU patients. Mortality was significantly higher in patients with haematological malignancy and intensive care. In addition, mortality was driven by comorbidities. Interestingly, the omicron variants of concern (VOC) were associated with no progression to critical COVID-19. Vaccination was the main factor to reduce the likelihood of severe COVID 19 and need for ICU.

High rates of severe illness and mortality have been recorded in this patient population [4, 7, 22]. Throughout the pandemic the risk to develop severe COVID-19 requiring intensive care was more than three times higher (16% versus 5%) in patients with haematological malignancies [7, 23–25]. The risk varied with the dominant VOC, with intensive care risk rates reaching 21.8% during the “second wave” among immunosuppressed patients. Although, the likelihood of developing severe COVID-19 requiring intensive care decreased from 5 to 0.1–0.2% in the immunocompetent population, patients with haematological malignancy still faced substantially higher intensive care admission rates (8.4%) [26, 27].

The COVID-19-associated average global mortality rate in a general population was 1.1% with a wide range between different countries [27]. Within critically ill patients with a variety of underlying conditions, ICU mortality reached 32.7%, which exceeded up to 73% in ventilated patients with need for renal replacement, then declined to 25% during the pandemic [28, 29]. This contrasts with mortality rates in our study of 25.4% in the entire population of patients with haematological malignancy and even 62.8% in the intensive care group with higher rates in patients facing active disease with mortality rates up to approximately 83.8%. With the emergence of the omicron variants, an overall milder clinical course and lower mortality rates (5.7% in patients with wild type virus, 0.86% in patients with omicron variants) in all patients were observed. This trend was also detected in the ICU mortality (10% of patients with omicron variants died vs. 32.7% of patients with wild type died) [26, 28, 30–32]. Although, the risk of critical infections decreased in immunocompetent patients, our study reports mortality rates in critically ill patients with haematological malignancy remained elevated, with 48.4% and 66.1% in patients with active haematological malignancy during the omicron wave.

The mortality decrease over subsequent COVID-19 waves may be attributed to advances in patient care ranging from vaccination to introduction of targeted COVID-19 treatments [33–35]. New insights into the SARS-CoV-2 virus resulted in variety of potential therapeutic options [35, 36]. Although, COVID-19 therapy was not explicitly investigated in our cohort, the early initiation of corticosteroids so as the introduction of tocilizumab and antibodies in the management of severe COVID-19, may have potentially improved the outcome of these patients. The justified fear of further immunodepression using targeted therapeutic approaches lessened over time that early initiation of such therapies may also be beneficial in patients with haematological malignancy [9, 34].

Comorbidities and active haematological malignancy were confirmed in the present study as negative prognostic factors that had not changed since the pre-vaccination era [7, 37]. Apart from the known risk factors for severe COVID-19 such as pre-existing lung disease, obesity or cardiopathy, the extent of immunodeficiency and iatrogenic immunosuppression may also have impacted overall prognosis [9, 27, 38]. Outcomes may differ depending on the underlying haematological malignancy, its activity, and its therapy [8, 10–13, 15, 39]. The most plausible variables that may impact outcome in our patients include hypogammaglobulinemia, qualitative and quantitative B- and T-cell deficiencies, CD4 + lymphopenia, innate immune dysfunction, and neutropenia, all resulting from haematological malignancy itself and respective treatment [9, 27, 38]. Notwithstanding these potential implications, the recently published recommendations from European Conference of Infections in Leukaemia (ECIL-9) underlined the crucial role of mRNA-based vaccines against COVID-19 and recommended their use in patients with haematological malignancy [23–25, 38, 40, 41]. According to our findings, immunisation may have contributed significantly to reduce the need for intensive care and mortality in patients with haematological malignancy. It is clear that more research into the impact of additional vaccine booster doses and prophylactic monoclonal antibody administration in patients with an ineffective response to vaccination is desirable, especially in patients with severe or critical COVID-19 [23–25, 36].

Despite our findings, we plead for ICU treatment of patients with hematological malignancy patients which is sometimes discussed with some reluctance between intensivists and hemato-oncologists. Not only in COVID-19-affected patients but also in hematological malignancy patients without COVID-19 the outcome of these patients if ICU is needed increased during the last years [42, 43].

Some limitations of our study are the retrospective observational design and the possible selection bias owing to the large number of participating institutions. Moreover, our results did not focus on therapeutic strategies while patients were on ICU, as these were not possible to retrieve. Further prospective studies may evaluate the role of intensive care therapies in patients with haematological malignancies. Additionally, the study's limitations include the absence of data regarding co-infections, pulmonary embolism, shock, and other management within the ICU, as well as the inability to incorporate information about the timing and reason of ICU admission after positive COVID-19 PCR and symptom onset. Besides, no data regarding organ support or failure beyond ventilation or complications were obtained, and information on the admission policy for severe patients within the ICU and COVID-19-related treatment limitations was also not accessible. In the latter case associated mainly to lack of treatment strategies at pandemic onset. Furthermore, despite the online survey within EPICOVIDEHA prompts patient contributors to furnish serological levels post-vaccination (and pre-COVID-19), the actual performance and timing of such tests depend on the internal policies of individual hospitals and healthcare agencies. Consequently, this information may be unavailable in many cases, and even when available, it can be often in a limited number of cases and not always before the COVID-19 diagnosis. Given these constraints and to ensure the meaningfulness of our findings, we opted not to include this information in our patient data.

The study reveals that the risk of severe COVID-19 requiring ICU admission has notably declined in vaccinated, non-immunocompromised population, especially with the emergence of the omicron variant. However, patients with haematological malignancies, particularly those with active disease, continue to face a heightened risk of severe COVID-19 and ICU admission. Factors like male sex, comorbidities, and specific COVID-19 symptoms increase the likelihood of ICU admission in these patients, while vaccination significantly reduces this risk. Mortality rates in ICU-managed patients with haematological malignancies remain substantially higher than those outside the ICU, influenced by factors like active malignancy, age, and comorbidities. The study underscores the importance of ongoing research, vaccination, and appropriate treatments to enhance outcomes for this high-risk patient group.

### Supplementary Information

Below is the link to the electronic supplementary material.

Supplementary file 1 (DOCX 461 kb)

## Data Availability

The data utilized in this study are accessible upon reasonable request to the corresponding author.

## Declarations

### Data sharing

Reasonable requests for data sharing may be submitted to Livio Pagano (livio.pagano@unicatt.it).
