# Correction: Need for ICU and outcome of critically ill patients with COVID-19 and haematological malignancies: results from the EPICOVIDEHA survey

**DOI:** 10.1007/s15010-024-02240-x

**Published:** 2024-05-21

**Authors:** Tobias Lahmer, Jon Salmanton-García, Francesco Marchesi, Shaimaa El-Ashwah, Marcio Nucci, Caroline Besson, Federico Itri, Ozren Jaksic, Natasha Čolović, Barbora Weinbergerová, Guldane Cengiz Seval, Tatjana Adžić-Vukičević, Tomáš Szotkowski, Uluhan Sili, Michelina Dargenio, Jens van Praet, Jaap van Doesum, Martin Schönlein, Zdeněk Ráčil, Pavel Žák, Christian Bjørn Poulsen, Gabriele Magliano, Moraima Jiménez, Valentina Bonuomo, Klára Piukovics, Giulia Dragonetti, Fatih Demirkan, Ola Blennow, Toni Valković, Maria Gomes Da Silva, Johan Maertens, Andreas Glenthøj, Noemí Fernández, Rui Bergantim, Luisa Verga, Verena Petzer, Ali S. Omrani, Gustavo-Adolfo Méndez, Marina Machado, Marie-Pierre Ledoux, Rebeca Bailén, Rafael F. Duarte, Maria Ilaria Del Principe, Francesca Farina, Sonia Martín-Pérez, Julio Dávila-Valls, Monia Marchetti, Yavuz M. Bilgin, Nicola S. Fracchiolla, Chiara Cattaneo, Ildefonso Espigado, Raul Cordoba, Graham P. Collins, Jorge Labrador, Iker Falces-Romero, Lucia Prezioso, Stef Meers, Francesco Passamonti, Caterina Buquicchio, Alberto López-García, Austin Kulasekararaj, Irati Ormazabal-Vélez, Annarosa Cuccaro, Carolina Garcia-Vidal, Alessandro Busca, Milan Navrátil, Nick de Jonge, Monika M. Biernat, Anna Guidetti, Ghaith Abu-Zeinah, Michail Samarkos, Amalia Anastasopoulou, Cristina de Ramón, Tomás José González-López, Martin Hoenigl, Olimpia Finizio, László Imre Pinczés, Natasha Ali, Antonio Vena, Carlo Tascini, Zlate Stojanoski, Maria Merelli, Ziad Emarah, Milena Kohn, Aleksandra Barać, Miloš Mladenović, Bojana Mišković, Osman Ilhan, Gökçe Melis Çolak, Martin Čerňan, Stefanie K. Gräfe, Emanuele Ammatuna, Michaela Hanakova, Benjamín Víšek, Alba Cabirta, Anna Nordlander, Raquel Nunes Rodrigues, Ditte Stampe Hersby, Giovanni Paolo Maria Zambrotta, Dominik Wolf, Lucía Núñez-Martín-Buitrago, Elena Arellano, Tommaso Francesco Aiello, Ramón García-Sanz, Juergen Prattes, Matthias Egger, Alessandro Limongelli, Martina Bavastro, Milche Cvetanoski, Miriam Dibos, Sebastian Rasch, Laman Rahimli, Oliver A. Cornely, Livio Pagano, the EPICOVIDEHA registry, Joseph Meletiadis, Florian Reizine, Jan Novák, Summiya Nizamuddin, Roberta Di Blasi, Alexandra Serris, Pavel Jindra, Sylvain Lamure, François Danion, Maria Chiara Tisi, Mario Virgilio Papa, Nurettin Erben, Ľuboš Drgoňa, Nathan C. Bahr, Murtadha Al-Khabori, Ayten Shirinova, Jörg Schubert, Lisset Lorenzo De La Peña, José-Ángel Hernández-Rivas, Elena Busch, Josip Batinić, Giuseppe Sapienza, Mohammad Reza Salehi, Reham Abdelaziz Khedr, Nina Khanna, Baerbel Hoell-Neugebauer, Ana Groh, Eleni Gavriilaki, Rita Fazzi, Rémy Duléry, Roberta Della Pepa, Mario Delia, Nicola Coppola, Maria Calbacho, Darko Antić, Hossein Zarrinfer, Ayel Yahia, Vivien Wai-Man, Ana Torres-Tienza, Alina Daniela Tanasa, Andrés Soto-Silva, Laura Serrano, Enrico Schalk, Ikhwan Rinaldi, Gaëtan Plantefeve, Monica Piedimonte, Maria Enza Mitra, Carolina Miranda-Castillo, Jorge Loureiro-Amigo, Ira Lacej, Martin Kolditz, María-Josefa Jiménez-Lorenzo, Guillemette Fouquet, Omar-Francisco Coronel-Ayala, Mathias Brehon, Panagiotis Tsirigotis, Anastasia Antoniadou, Gina Varricchio, Maria Vehreschild, Agostino Tafuri, José-María Ribera-Santa Susana, Joyce Marques De Almeida, María Fernández-Galán, Avinash Aujayeb, Athanasios Tragiannidis, Malgorzata Mikulska, Sein Win, Elizabeth De Kort, Hans-Beier Ommen, Donald C. Vinh, Hans Martin Orth, Sandra Malak, Przemyslaw Zdziarski, Modar Saleh, Chi Shan Kho, Fabio Guolo, M. Mansour Ceesay, Christopher H. Heath, Sergey Gerasymchuk, Monica Fung, Maximilian Desole, Erik De Cabo, Tania Cushion, Fazle Rabbi Chowdhury, Louis Yi Ann Chai, Fevzi Altuntaş, Charlotte Flasshove

**Affiliations:** 1 Medizinische Klinik II, Klinikum rechts der Isar, TU München, Munich, Germany; 2 Faculty of Medicine, and University Hospital Cologne, Institute of Translational Research, Cologne Excellence Cluster on Cellular Stress Responses in Aging-Associated Diseases (CECAD), University of Cologne, Herderstraße 52-54, 50931 Cologne, Germany; 3 Faculty of Medicine, University Hospital Cologne, Department I of Internal Medicine, Center for Integrated Oncology Aachen Bonn Cologne Duesseldorf (CIO ABCD) and Excellence Center for Medical Mycology (ECMM), University of Cologne, Cologne, Germany; 4 German Centre for Infection Research (DZIF), Partner Site Bonn-Cologne, Cologne, Germany; 5 Hematology and Stem Cell Transplant Unit, IRCCS Regina Elena National Cancer Institute, Rome, Italy; 6 Oncology Center, Mansoura University, Mansoura, Egypt; 7 Federal University of Rio de Janeiro, Rio de Janeiro, Brazil; 8 Centre Hospitalier de Versailles, Le Chesnay, France; Université Paris-Saclay, UVSQ, Inserm, Équipe “Exposome et Hérédité”, CESP, Villejuif, France; 9 San Luigi Gonzaga Hospital - Orbassano, Orbassano, Italy; 10 Department of Hematology, University Hospital Dubrava, Zagreb, Croatia; 11 University Clinical Center Serbia, Medical Faculty University Belgrade, Belgrade, Serbia; 12 Department of Internal Medicine - Hematology and Oncology, Masaryk University Hospital Brno, Brno, Czech Republic; 13 Ankara University, Ankara, Turkey; 14 COVID hospital “Batajnica”, Belgrade, Serbia; 15 University Hospital Olomouc, Olomouc, Czech Republic; 16 Department of Infectious Diseases and Clinical Microbiology, School of Medicine, Marmara University, Istanbul, Turkey; 17 Hematology and Stem Cell Transplan Unit, Vito Fazzi Hospital, Lecce, Italy; 18 Department of Nephrology and Infectious Diseases, AZ Sint-Jan Brugge-Oostende AV, Brugge, Belgium; 19 University Medical Center Groningen, Groningen, Netherlands; 20 Department of Oncology, Hematology and Bone Marrow Transplantation with Section of Pneumology, University Medical Center Hamburg-Eppendorf, Hamburg, Germany; 21 Institute of Hematology and Blood Transfusion, Prague, Czech Republic; 22 University Hospital Hradec Králové, Hradec Králové, Czech Republic; 23 Zealand University Hospital, Roskilde, Roskilde, Denmark; 24 ASST Grande Ospedale Metropolitano Niguarda, Milan, Italy; 25 Department of Hematology, Vall d’Hebron Hospital Universitari, Experimental Hematology, Vall d’Hebron Institute of Oncology (VHIO), Vall d’Hebron Barcelona Hospital Campus, Barcelona, Spain; 26 Departament de Medicina, Universitat Autònoma de Barcelona, Bellaterra, Spain; 27 Department of Medicine, Section of Hematology, University of Verona, Verona, Italy; 28 Department of Clinical and Biological Sciences, University of Turin, Turin, Italy; 29 Department of Internal Medicine, South Division Faculty of Medicine, University of Szeged, Szeged, Hungary; 30 Hematology Unit, Fondazione Policlinico Universitario Agostino Gemelli - IRCCS, Rome, Italy; 31 Division of Hematology, Dokuz Eylul University, Izmir, Turkey; 32 Department of Infectious Diseases, Karolinska University Hospital, Stockholm, Sweden; 33 University Hospital Centre Rijeka, Rijeka, Croatia; 34 Croatian Cooperative Group for Hematological Diseases (CROHEM), Faculty of Medicine and Faculty of Health Studies, University of Rijeka, Rijeka, Croatia; 35 Portuguese Institute of Oncology, Lisbon, Portugal; 36 Department of Microbiology, Immunology, and Transplantation, KULeuven, Leuven and Department of Hematology, UZ Leuven, Louvain, Belgium; 37 Department of Hematology, Copenhagen University Hospital - Rigshospitalet, Copenhagen, Denmark; 38 Hospital Universitario Marqués de Valdecilla, Santander, Spain; 39 Centro Hospitalar e Universitário São João, Porto, Portugal; 40 Azienda Ospedaliera San Gerardo - Monza, Monza, Italy; 41 Università Milano-Bicocca, Milan, Italy; 42 Department of Hematology and Oncology, Comprehensive Cancer Center Innsbruck (CCCI), Medical University of Innsbruck (MUI), Innsbruck, Austria; 43 Communicable Disease Center, Hamad Medical Corporation, Doha, Qatar; 44 Hospital Escuela de Agudos Dr. Ramón Madariaga, Posadas, Argentina; 45 Clinical Microbiology and Infectious Diseases Department, Hospital General Universitario Gregorio Marañón, Madrid, Spain; 46 ICANS, Strasbourg, France; 47 Hematology Department, Hospital General Universitario Gregorio Marañón, Madrid, Spain; 48 Hospital Universitario Puerta de Hierro, Majadahonda, Spain; 49 Hematology, Department of Biomedicine and Prevention, University of Rome Tor Vergata, Rome, Italy; 50 IRCCS Ospedale San Raffaele, Milan, Italy; 51 Hospital Nuestra Señora de Sonsoles, Ávila, Spain; 52 Azienda Ospedaliera Nazionale SS. Antonio e Biagio e Cesare Arrigo, Alessandria, Italy; 53 Department of Internal Medicine, ADRZ, Goes, Netherlands; 54 Fondazione IRCCS Ca’ Granda Ospedale Maggiore Policlinico, Milan, Italy; 55 Hematology Unit, ASST-Spedali Civili, Brescia, Italy; 56 Department of Hematology, University Hospital Virgen Macarena - University Hospital Virgen del Rocío, Instituto de Biomedicina de Sevilla (IBIS / CSIC), Universidad de Sevilla (Departamento de Medicina), Seville, Spain; 57 Fundacion Jimenez Diaz University Hospital, Health Research Institute IIS-FJD, Madrid, Spain; 58 NIHR Oxford Biomedical Research Centre, Churchill Hospital, Oxford, UK; 59 Department of Hematology, Research Unit, Hospital Universitario de Burgos, Burgos, Spain; 60 Facultad de Ciencias de la Salud, Universidad Isabel I, Burgos, Spain; 61 La Paz University Hospital, Madrid, Spain; 62 CIBERINFEC, Instituto de Salud Carlos III, Madrid, Spain; 63 Hospital University of Parma - Hematology and Bone Marrow Unit, Parma, Italy; 64 AZ KLINA, Brasschaat, Belgium; 65 Department of Medicine and Surgery, University of Insubria and ASST Sette Laghi, Ospedale di Circolo of Varese, Varese, Italy; 66 Ematologia con Trapianto, Ospedale Dimiccoli Barletta, Barletta, Italy; 67 King’s College Hospital, London, UK; 68 King’s College London, London, UK; 69 Complejo Hospitalario de Navarra, Iruña-Pamplona, Spain; 70 Hematology Unit, Center for Translational Medicine, Azienda USL Toscana NordOvest, Leghorn, Italy; 71 Hospital Clinic, Barcelona, Spain; 72 Stem Cell Transplant Center, AOU Citta’ della Salute e della Scienza, Turin, Italy; 73 Head of the ICU and Transplant Unit, Department of Hematooncology, University Hospital of Ostrava, Ostrava-Poruba, Czech Republic; 74 Amsterdam UMC, location VUmc, Amsterdam, Netherlands; 75 Department of Haematology, Blood Neoplasms, and Bone Marrow Transplantation, Wroclaw Medical University, Wroclaw, Poland; 76 University of Milan and Fondazione IRCCS Istituto Nazionale Dei Tumori, Milan, Italy; 77 Division of Hematology and Oncology, Weill Cornell Medicine, New York, USA; 78 Laikon Hospital, Athens, Greece; 79 Hematology Department, Hospital Universitario de Salamanca, Salamanca, Spain; 80 IBSAL, Centro de Investigación del Cáncer-IBMCC (USAL-CSIC), Salamanca, Spain; 81 Department of Hematology, Hospital Universitario de Burgos, Burgos, Spain; 82 Division of Infectious Diseases, Department of Internal Medicine, Medical University of Graz, Graz, Austria; 83 BioTechMed, Graz, Austria; 84 UOC Hematology, AORN Cardarelli, Naples, Italy; 85 Division of Hematology, Department of Internal Medicine, Faculty of Medicine, University of Debrecen, Debrecen, Hungary; 86 Aga Khan University, Karachi, Pakistan; 87 Ospedale Policlinico San Martino, Genoa, Italy; 88 Azienda Sanitaria Universitaria del Friuli Centrale, Udine, Italy; 89 University Clinic of Hematology, Skopje, North Macedonia; 90 Centre Hospitalier de Versailles, Versailles, France; 91 Clinic for Infectious and Tropical Diseases, University Clinical Center of Serbia, Faculty of Medicine, University of Belgrade, Belgrade, Serbia; 92 COVID hospital ““Batajnica””, Belgrade, Serbia; 93 Clinic for Orthopedic Surgery and Traumatology, University Clinical Center of Serbia, Belgrade, Serbia; 94 Center for Radiology, University Clinical Center of Serbia, Belgrade, Serbia; 95 Department of Hemato-Oncology, Faculty of Medicine and Dentistry, Palacky University and University Hospital Olomouc, Olomouc, Czech Republic; 96 Department of Medicine, University Medical Center Hamburg-Eppendorf, Hamburg, Germany; 97 Department of Hematology and Oncology, Medical University of Innsbruck, Innsbruck, Austria; 98 Head of Molecular Biology an HLA Unit, Department of Hematology, University Hospital of Salamanca (HUS/IBSAL/CIBERONC), Salamanca, Spain; 99 Medical University of Graz, Graz, Austria; 100 Faculty of Medicine, and University Hospital Cologne, Clinical Trials Centre Cologne (ZKS Köln), University of Cologne, Cologne, Germany; 101 Faculty of Medicine, and University Hospital Cologne, Center for Molecular Medicine Cologne (CMMC), University of Cologne, Cologne, Germany; 102 Hematology Unit, Università Cattolica del Sacro Cuore, Rome, Italy

**Correction: Infection** 10.1007/s15010-023-02169-7

**Acknowledgements** Members of the EPICOVIDEHA registry: Joseph Meletiadis, Florian Reizine, Jan Novák, Summiya Nizamuddin, Roberta Di Blasi, Alexandra Serris, Pavel Jindra, Sylvain Lamure, François Danion, Maria Chiara Tisi, Mario Virgilio Papa, Nurettin Erben, ľuboš DrgoňA, Nathan C. Bahr, Murtadha Al-Khabori, Ayten Shirinova, Jörg Schubert, Lisset Lorenzo De La Peña, José-Ángel Hernández-Rivas, Elena Busch, Josip Batinić, Giuseppe Sapienza, Mohammad Reza Salehi, Reham Abdelaziz Khedr, Nina Khanna, Baerbel Hoell-Neugebauer, Ana Groh, Eleni Gavriilaki, Rita Fazzi, Rémy Duléry, Roberta Della Pepa, Mario Delia, Nicola Coppola, Maria Calbacho, Darko Antić, Hossein Zarrinfer, Ayel Yahia, Vivien Wai-Man, Ana Torres-TIenza, Alina Daniela Tanasa, Andrés Soto-Silva, Laura Serrano, Enrico Schalk, Ikhwan Rinaldi, Gaëtan Plantefeve, Monica Piedimonte, Maria Enza Mitra, Carolina Miranda-Castillo, Jorge Loureiro-Amigo, Ira Lacej, Martin Kolditz, María-Josefa Jiménez-Lorenzo, Guillemette Fouquet, Omar-Francisco Coronel-Ayala, Mathias Brehon, Panagiotis Tsirigotis, Anastasia Antoniadou, Gina Varricchio, Maria Vehreschild, Agostino Tafuri, José-María Ribera-Santa Susana, Joyce Marques De Almeida, María Fernández-Galán, Avinash Aujayeb, Athanasios Tragiannidis, Malgorzata Mikulska, Sein Win, Elizabeth De Kort, Hans-Beier Ommen, Donald C. Vinh, Hans Martin Orth, Sandra Malak, Przemyslaw Zdziarski, Modar Saleh, Chi Shan Kho, Fabio Guolo, M. Mansour Ceesay, Christopher H. Heath, Sergey Gerasymchuk, Monica Fung, Maximilian Desole, Erik De Cabo, Tania Cushion, Fazle Rabbi Chowdhury, Louis Yi Ann Chai, Fevzi Altuntaş, Charlotte Flasshove.

The original article has been updated.

