# Supplementary material for: Need for ICU and outcome of critically ill patients with COVID-19 and haematological malignancies: results from the EPICOVIDEHA survey
Source: Infection. 2024 Feb 22;52(3):1125–41. doi: 10.1007/s15010-023-02169-7 (PMC11143019; doi:10.1007/s15010-023-02169-7)
Supplement: Supplementary file 1 — (DOCX 461 kb) [file 15010_2023_2169_MOESM1_ESM.docx]

**NEED FOR ICU AND OUTCOME OF CRITICALLY ILL PATIENTS WITH COVID-19 AND HAEMATOLOGICAL MALIGNANCIES: RESULTS FROM THE EPICOVIDEHA SURVEY**

**Supplementary figures**

**Supplementary figure 1. Survival probability of patients with haematological malignancy and COVID-19, by need for intensive care and previous vaccination and country (for countries with n>100).**

| 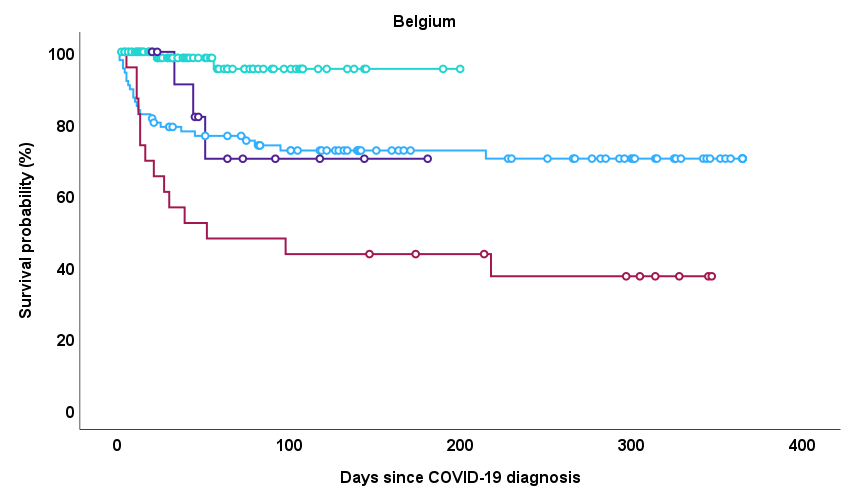 | \| **<0.001** \| **No ICU no vax** \| **ICU no vax** \| **No ICU vax** \| \| --- \| --- \| --- \| --- \| \| **ICU no vax** \| **0.009** \|  \|  \| \| **No ICU vax** \| **<0.001** \| **<0.001** \|  \| \| **ICU vax** \| 0.814 \| 0.088 \| **0.014** \| |
| --- | --- | --- | --- | --- | --- | --- | --- | --- | --- | --- | --- | --- | --- | --- | --- | --- | --- |
| 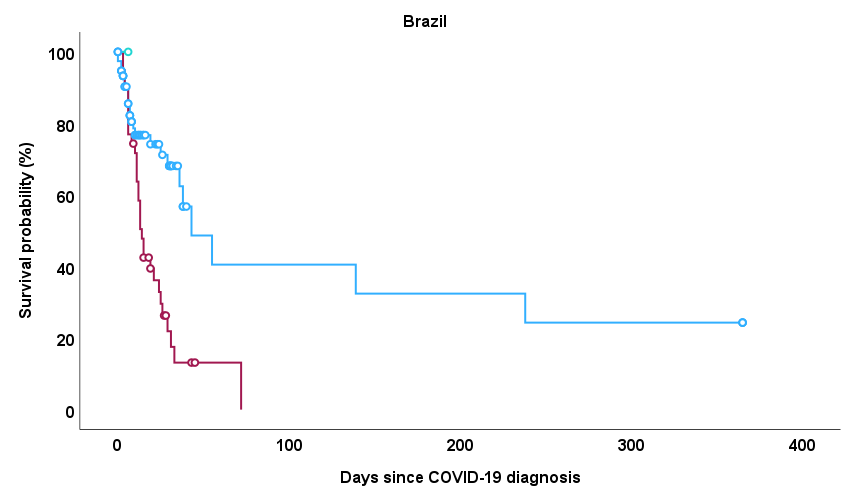 | \| **<0.001** \| **No ICU no vax** \| **ICU no vax** \| \| --- \| --- \| --- \| \| **ICU no vax** \| **<0.001** \|  \| \| **No ICU vax** \| 0.692 \| 0.604 \| |
| 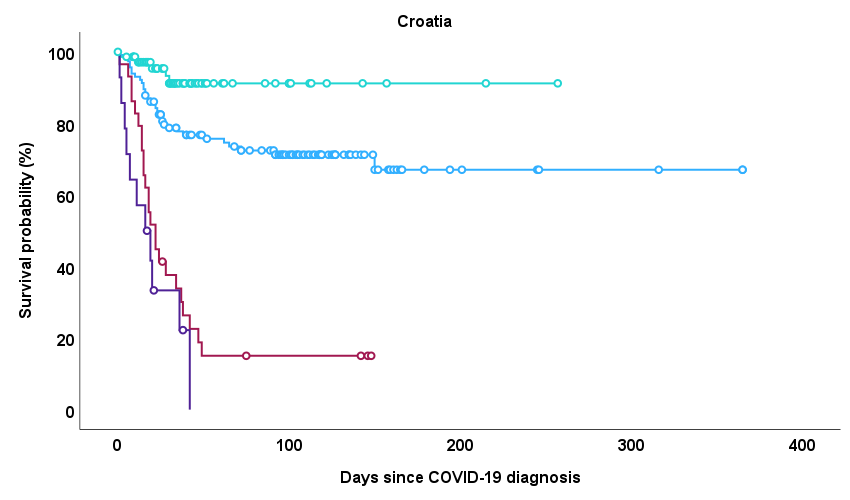 | \| **<0.001** \| **No ICU no vax** \| **ICU no vax** \| **No ICU vax** \| \| --- \| --- \| --- \| --- \| \| **ICU no vax** \| **<0.001** \|  \|  \| \| **No ICU vax** \| **0.010** \| **<0.001** \|  \| \| **ICU vax** \| **<0.001** \| 0.245 \| **<0.001** \| |
| 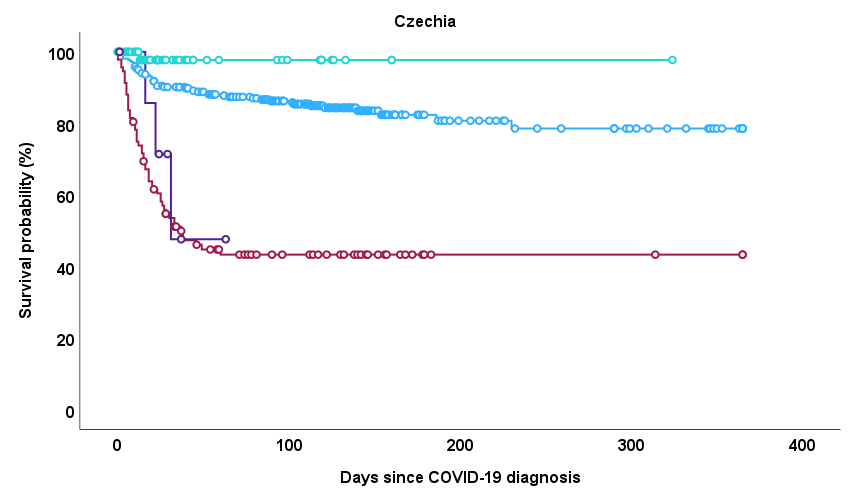 | \| **<0.001** \| **No ICU no vax** \| **ICU no vax** \| **No ICU vax** \| \| --- \| --- \| --- \| --- \| \| **ICU no vax** \| **<0.001** \|  \|  \| \| **No ICU vax** \| 0.058 \| **<0.001** \|  \| \| **ICU vax** \| **0.008** \| 0.590 \| **<0.001** \| |
| 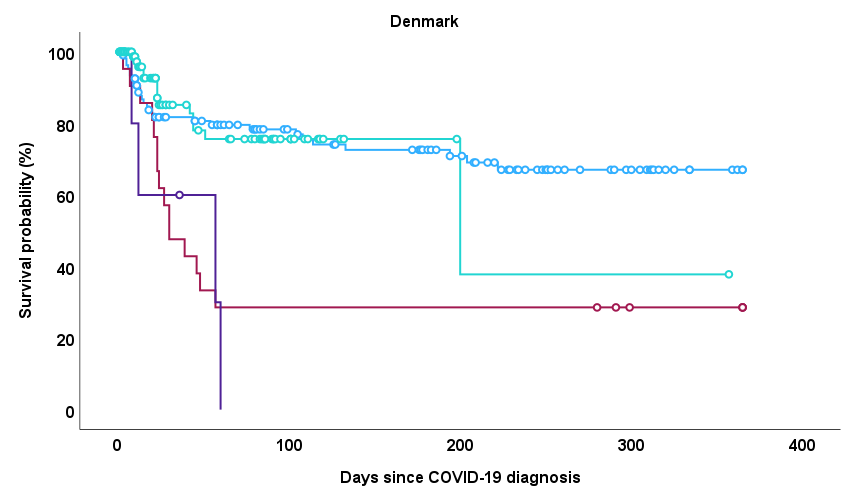 | \| **<0.001** \| **No ICU no vax** \| **ICU no vax** \| **No ICU vax** \| \| --- \| --- \| --- \| --- \| \| **ICU no vax** \| **<0.001** \|  \|  \| \| **No ICU vax** \| 0.868 \| **<0.001** \|  \| \| **ICU vax** \| **0.001** \| 0.724 \| **0.001** \| |
| 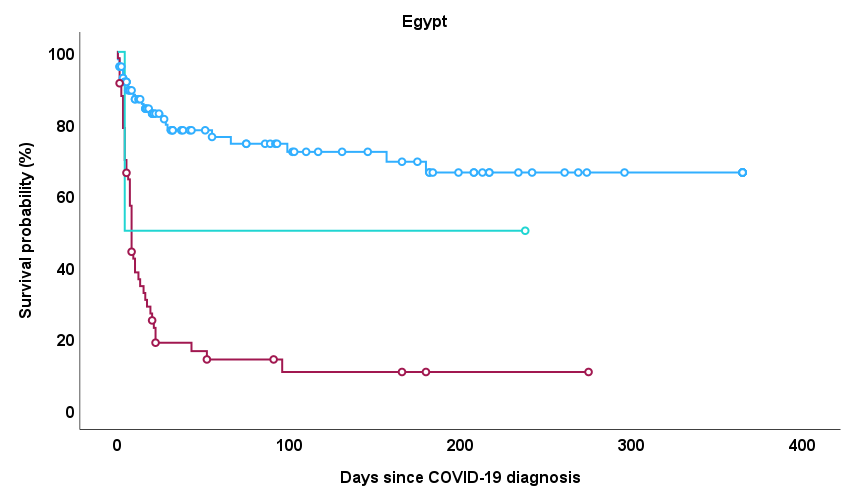 | \| **<0.001** \| **No ICU no vax** \| **ICU no vax** \| \| --- \| --- \| --- \| \| **ICU no vax** \| **<0.001** \|  \| \| **No ICU vax** \| 0.479 \| 0.358 \| |
| 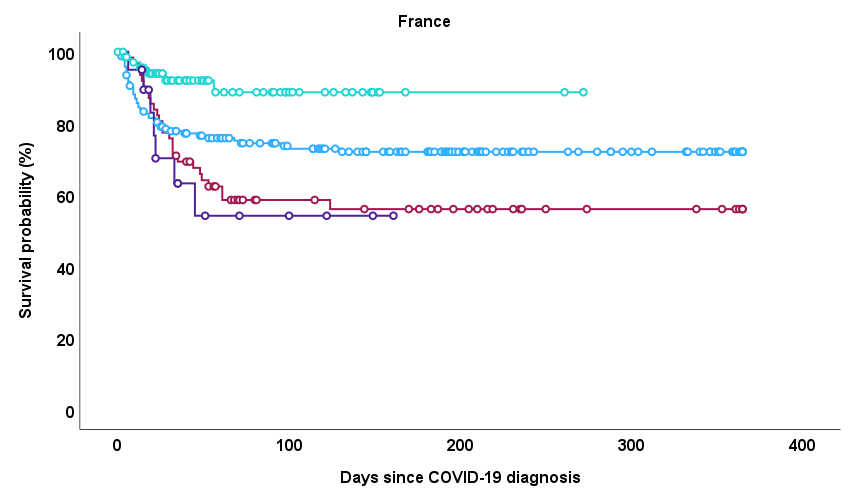 | \| **0.003** \| **No ICU no vax** \| **ICU no vax** \| **No ICU vax** \| \| --- \| --- \| --- \| --- \| \| **ICU no vax** \| 0.070 \|  \|  \| \| **No ICU vax** \| **0.011** \| **0.001** \|  \| \| **ICU vax** \| 0.292 \| 0.677 \| **0.002** \| |
| 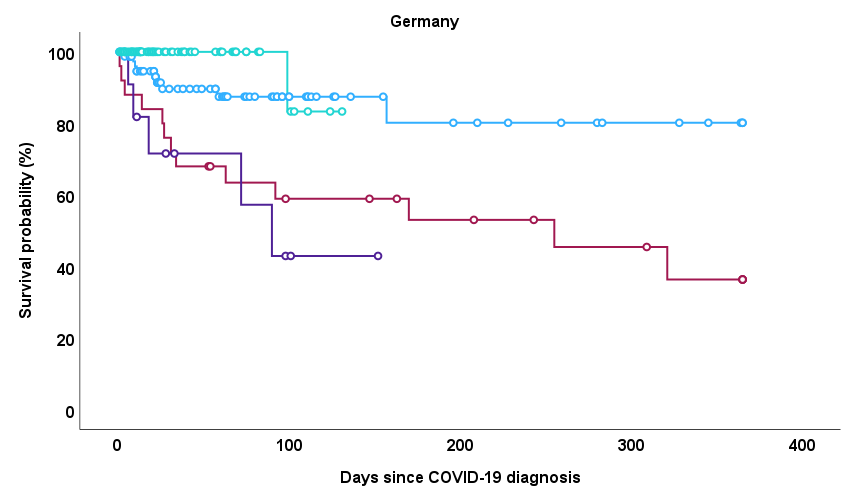 | \| **<0.001** \| **No ICU no vax** \| **ICU no vax** \| **No ICU vax** \| \| --- \| --- \| --- \| --- \| \| **ICU no vax** \| **0.003** \|  \|  \| \| **No ICU vax** \| 0.100 \| **0.001** \|  \| \| **ICU vax** \| **0.003** \| 0.562 \| **0.001** \| |
| 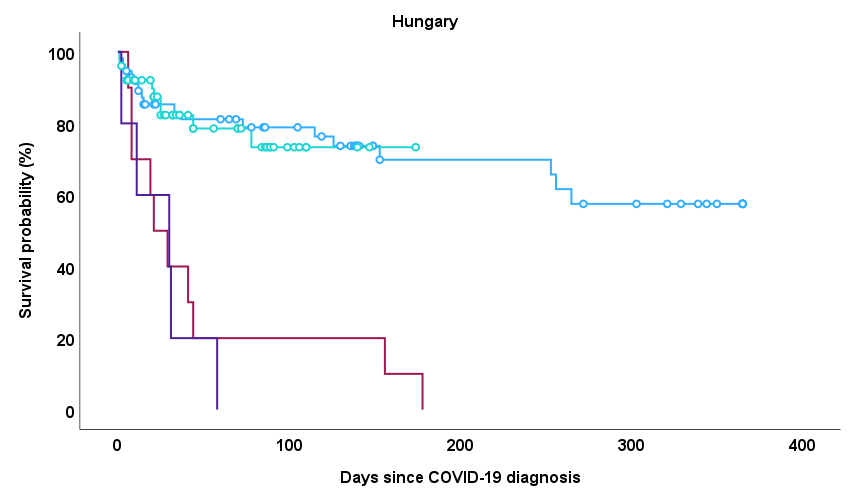 | \| **<0.001** \| **No ICU no vax** \| **ICU no vax** \| **No ICU vax** \| \| --- \| --- \| --- \| --- \| \| **ICU no vax** \| **<0.001** \|  \|  \| \| **No ICU vax** \| 0.806 \| **<0.001** \|  \| \| **ICU vax** \| **<0.001** \| 0.609 \| **<0.001** \| |
| 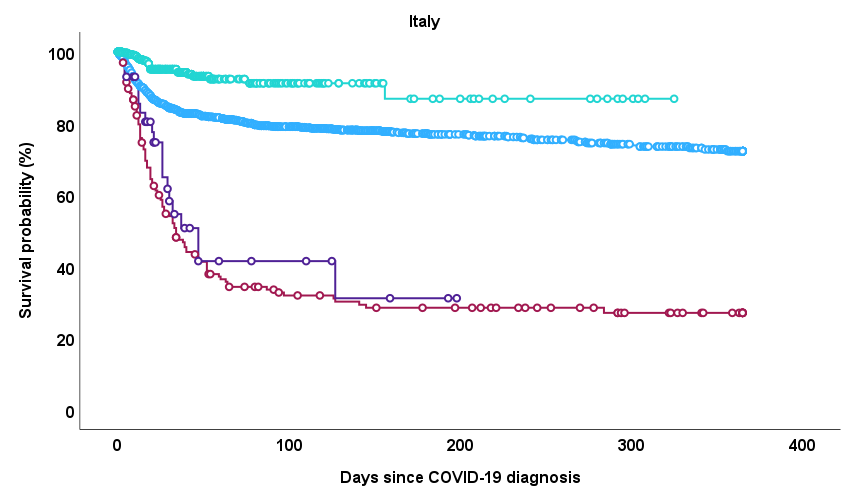 | \| **<0.001** \| **No ICU no vax** \| **ICU no vax** \| **No ICU vax** \| \| --- \| --- \| --- \| --- \| \| **ICU no vax** \| **<0.001** \|  \|  \| \| **No ICU vax** \| **<0.001** \| **<0.001** \|  \| \| **ICU vax** \| **<0.001** \| 0.347 \| **<0.001** \| |
| 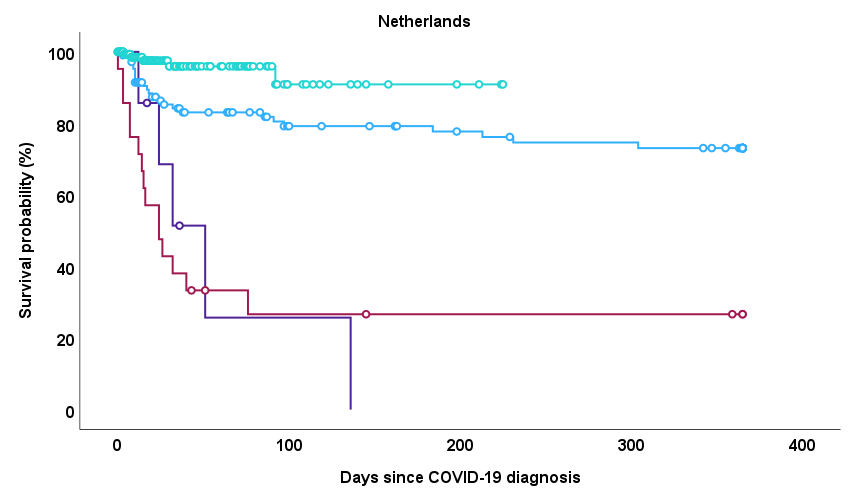 | \| **<0.001** \| **No ICU no vax** \| **ICU no vax** \| **No ICU vax** \| \| --- \| --- \| --- \| --- \| \| **ICU no vax** \| **<0.001** \|  \|  \| \| **No ICU vax** \| **0.006** \| **<0.001** \|  \| \| **ICU vax** \| **0.001** \| 0.821 \| **<0.001** \| |
| 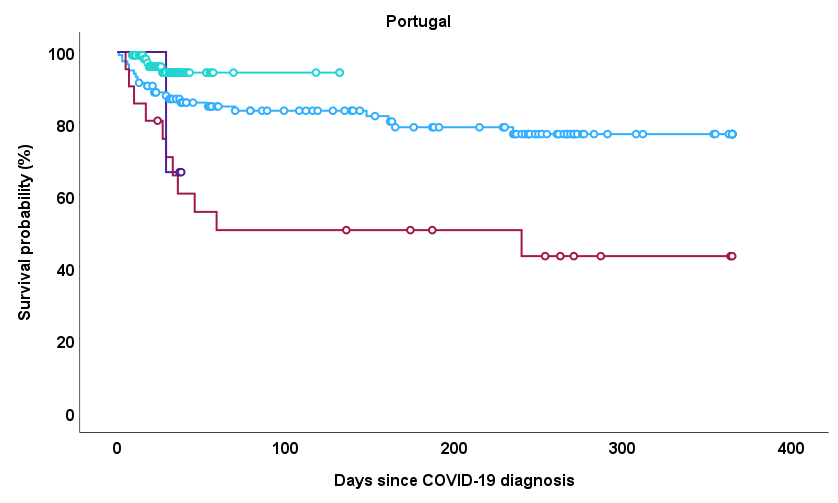 | \| **<0.001** \| **No ICU no vax** \| **ICU no vax** \| **No ICU vax** \| \| --- \| --- \| --- \| --- \| \| **ICU no vax** \| **0.001** \|  \|  \| \| **No ICU vax** \| 0.046 \| **<0.001** \|  \| \| **ICU vax** \| 0.385 \| 0.785 \| 0.101 \| |
| 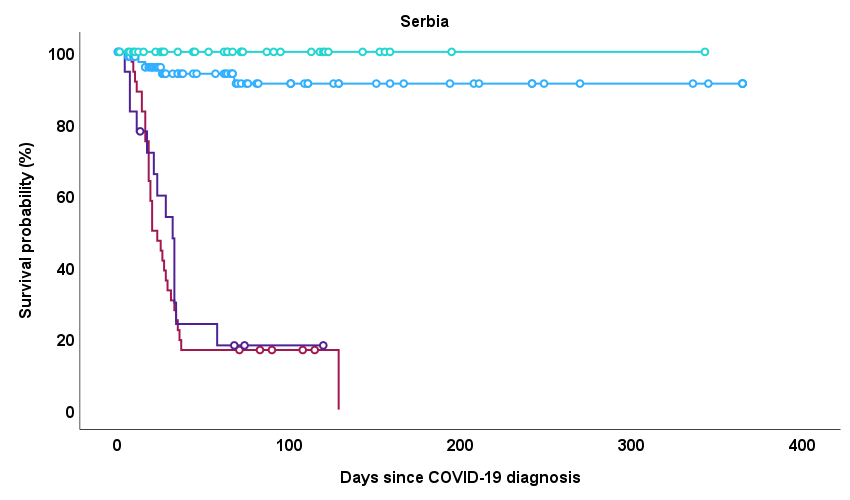 | \| **<0.001** \| **No ICU no vax** \| **ICU no vax** \| **No ICU vax** \| \| --- \| --- \| --- \| --- \| \| **ICU no vax** \| **<0.001** \|  \|  \| \| **No ICU vax** \| 0.123 \| **<0.001** \|  \| \| **ICU vax** \| **<0.001** \| 0.625 \| **<0.001** \| |
| 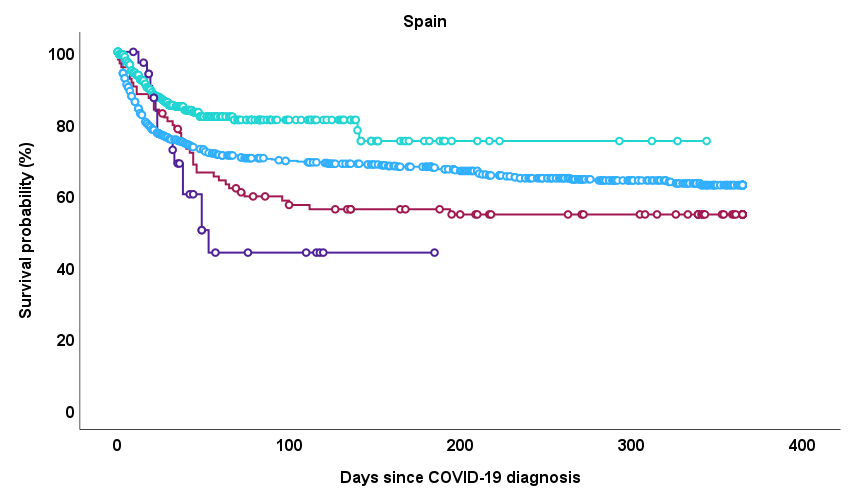 | \| **<0.001** \| **No ICU no vax** \| **ICU no vax** \| **No ICU vax** \| \| --- \| --- \| --- \| --- \| \| **ICU no vax** \| 0.159 \|  \|  \| \| **No ICU vax** \| **<0.001** \| **<0.001** \|  \| \| **ICU vax** \| 0.161 \| 0.284 \| **0.001** \| |
| 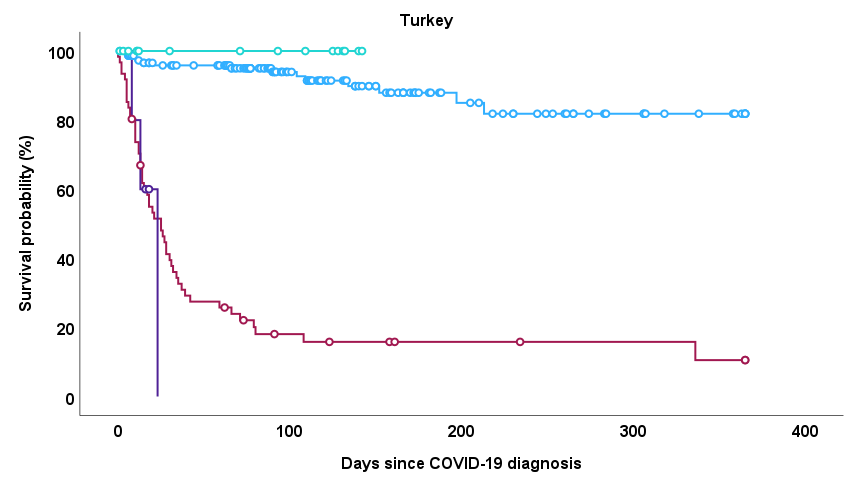 | \| **<0.001** \| **No ICU no vax** \| **ICU no vax** \| **No ICU vax** \| \| --- \| --- \| --- \| --- \| \| **ICU no vax** \| **<0.001** \|  \|  \| \| **No ICU vax** \| 0.338 \| **<0.001** \|  \| \| **ICU vax** \| **<0.001** \| 0.661 \| **0.001** \| |
| 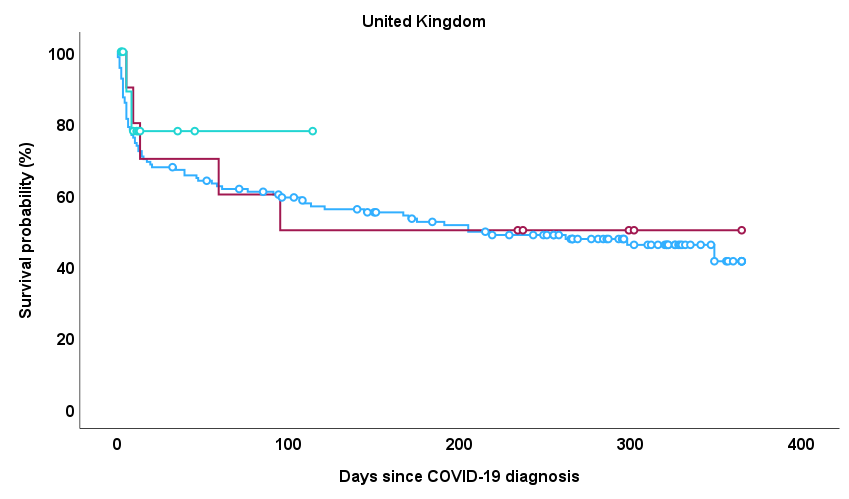 | \| 0.753 \| **No ICU no vax** \| **ICU no vax** \| \| --- \| --- \| --- \| \| **ICU no vax** \| 0.798 \|  \| \| **No ICU vax** \| 0.466 \| 0.702 \| |
